# Traumatic Posterior Sternoclavicular Joint Dislocation With a Five-Day Delayed Presentation: A Case Report

**DOI:** 10.7759/cureus.75271

**Published:** 2024-12-07

**Authors:** Salem Althuwaykh, Ahmed Alghamdi

**Affiliations:** 1 Orthopedic Department, King Fahad Medical City, Riyadh, SAU

**Keywords:** dislocation, posterior sternoclavicular joint dislocation, sternoclavicular, sternoclavicular joint, sternoclavicular joint dislocation

## Abstract

Posterior sternoclavicular joint (SCJ) dislocation is a rare but potentially life-threatening injury due to its proximity to critical mediastinal structures. Early diagnosis and prompt management are essential to prevent severe complications such as vascular or respiratory compromise. We report a case of a 23-year-old male who presented to our emergency department five days after a high-energy motor vehicle accident with isolated, closed posterior dislocation of the SCJ. The patient complained of left shoulder pain, mild dysphagia, and intermittent tingling in the left upper limb. Imaging revealed a posterior SCJ dislocation with minimal compression of the left brachiocephalic vein but no vascular injury. Closed reduction under general anesthesia was successfully performed using a towel clamp for manipulation. Stability was confirmed intraoperatively with fluoroscopy, and the patient was discharged 24 hours post procedure. Follow-up at two weeks and two months showed maintained reduction, resolution of symptoms, and full range of motion. In conclusion, posterior SCJ dislocations, while uncommon, require a high index of suspicion due to their potential for severe complications. Closed reduction remains a feasible treatment option even in delayed presentations up to five days post injury. The involvement of a multidisciplinary team is crucial during the management of these injuries to ensure comprehensive care and mitigate the risks associated with potential complications.

## Introduction

The sternoclavicular joint (SCJ) is a diarthrodial saddle-type joint that connects the medial end of the clavicle to the clavicular notch of the manubrium of the sternum and the first costal cartilage. It serves as the only bony articulation between the upper extremities and the axial skeleton, allowing for a range of movements and providing stability to the shoulder girdle [1,2].

Posterior dislocation of the SCJ is a rare but clinically significant injury, often resulting from high-energy trauma such as motor vehicle accidents or sports injuries. This type of dislocation poses a risk of serious complications due to the proximity of the joint to vital mediastinal structures. Complications can include respiratory distress, vascular injuries, brachial plexopathy, pneumothorax, dysphagia, and even death [3-5]. The need for prompt recognition and management of posterior dislocations is critical to prevent these life-threatening outcomes [6].

The literature emphasizes the need for prompt recognition and management of posterior dislocations to prevent life-threatening complications [7]. Overall, while posterior SCJ dislocations are uncommon, their potential for serious complications necessitates a high index of suspicion and timely intervention.

## Case presentation

Introduction

A 23-year-old male, a smoker and not known to have any medical disease, was referred to our emergency department from a local hospital following a motor vehicle accident (MVA) that occurred five days before his presentation. The accident involved a high-speed collision with another vehicle and subsequent rollover. He presented with isolated, closed posterior sternoclavicular dislocation.

Presentation

The patient’s primary complaint was left shoulder pain, predominantly at the SCJ, accompanied by intermittent tingling sensations in the left upper limb. These sensory changes did not follow a specific dermatome and were exacerbated by lying in the supine position. Additionally, the patient reported mild dysphagia but denied any breathing difficulties, dysphonia, or cervical pain.

Physical examination

On examination, the patient was vitally stable with no visible skin changes or signs of venous congestion in the arm or neck. Slight asymmetrical swelling was noted on the left medial clavicle. The shoulder's range of motion was limited due to pain. Neurological examination revealed normal myotome and dermatome distributions, and the axillary nerve and distal neurovascular examination was unremarkable.

Imaging studies

**Figure 1:**
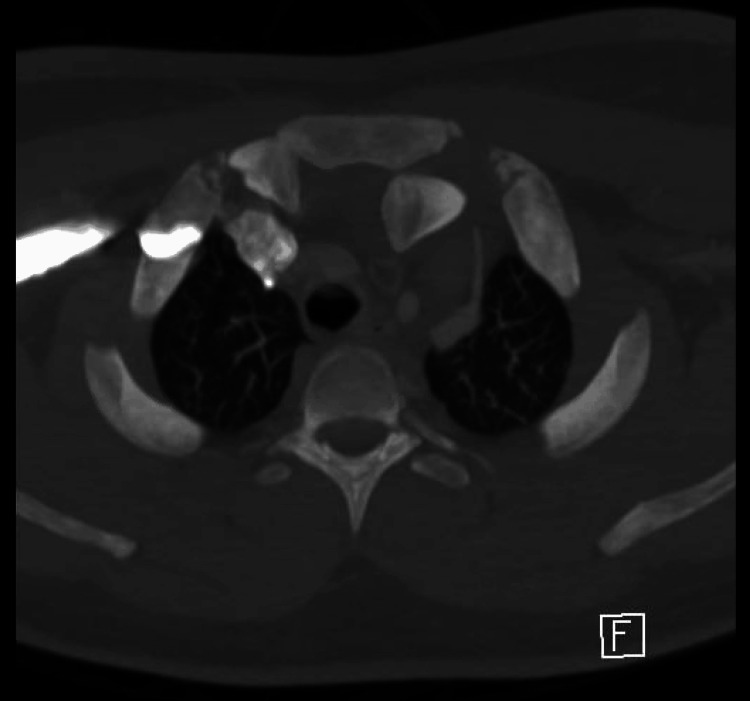
Axial chest CT angiography showing posterior dislocation of the left sternoclavicular joint.

Initial radiographs demonstrated asymmetry at the left SCJ. A subsequent CT scan confirmed a posterior dislocation of the left SCJ with subluxation of the right joint. CT angiography revealed no evidence of arterial vascular injury, and the posterior dislocation of the left SCJ minimally compressed the left brachiocephalic vein without obstruction (Figure 1).

Treatment and procedure

The decision was made to proceed with closed reduction under general anesthesia, with thoracic and vascular surgery backup available. The patient was placed under general anesthesia, and the anesthesia team inserted an arterial line and two peripheral IV lines. Six units of packed red blood cells were prepared on standby in case of intraoperative complications.

The patient was positioned supine with a bolster placed interscapularly. The surgical field was prepped and draped from the neck to the umbilicus. Baseline X-rays were obtained, including a serendipity view at the level of the SCJ with a 40-degree cephalic angle as well as an anteroposterior view, to assess the dislocation.

The reduction was performed by abducting the shoulder to 90 degrees and extending it by 20 degrees. A towel clamp was inserted percutaneously onto the proximal third of the clavicle, and the clavicle was then manipulated anteriorly and superiorly. A distinct “clunk” was felt, indicating successful reduction. Clinical examination and intraoperative fluoroscopy confirmed that the dislocation had been reduced. Following the reduction, stability was assessed by applying the towel clamp and manually attempting to move the clavicle. These maneuvers demonstrated a stable reduction with no further displacement observed.

Post-reduction, the patient was immobilized with a figure-eight bandage and an arm sling. A follow-up CT scan was performed in the radiology department, confirming the successful reduction. The patient remained stable and was monitored for 24 hours before being discharged with instructions for follow-up care.

Follow-up

**Figure 2:**
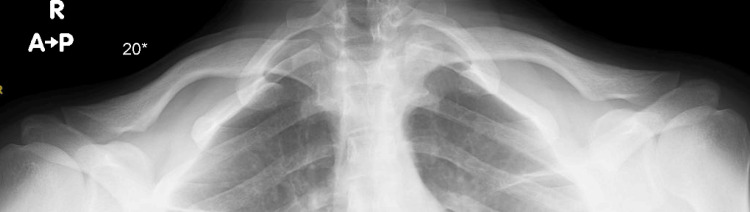
Anteroposterior-angled cephalic 20-degree X-ray of the bilateral clavicle done three months postoperatively showed reduced and symmetrical sternoclavicular joint.

At the two-week postoperative clinic visit, the reduction remained stable, with the patient reporting significant pain relief and the resolution of tingling sensations and dysphagia. At the two-month and three-month follow-up, the patient was pain-free, with a full range of motion in the left shoulder. Radiographic imaging showed a reduced, symmetrical SCJ (Figure 2).

## Discussion

The existing literature indicates that traumatic posterior dislocations of the SCJ are rare injuries, accounting for less than 1% of all dislocations [6]. These injuries are often associated with high-energy trauma, such as motor vehicle accidents or sports injuries, and can be challenging to diagnose due to their subtle presentation [6,8].

The complications arising from posterior SCJ dislocations are significant and can include serious conditions such as respiratory distress, vascular injuries, brachial plexopathy, pneumothorax, dysphagia, and even death. The proximity of the medial clavicle to vital mediastinal structures, including the trachea, esophagus, and major blood vessels, heightens the risk of these complications [9,10]. Studies have shown that up to 30% of patients with posterior dislocations may experience complications related to mediastinal compression [3,11,12].

The rarity of these injuries often leads to delayed diagnosis, which can exacerbate the risk of severe outcomes. Standard radiographs often fail to adequately visualize these injuries, necessitating the use of advanced imaging modalities such as CT to confirm the diagnosis and assess for associated injuries [13-15]. The use of CT angiography is particularly recommended to evaluate the vascular anatomy and identify any potential compromise [14]. This emphasizes the necessity for a high index of suspicion and the importance of thorough imaging in cases of suspected SCJ dislocation, especially in the context of high-energy trauma [11].

Management strategies for posterior SCJ dislocations have evolved, with both closed and open reduction techniques being employed. The literature suggests that for acute, traumatic SCJ dislocations, a closed reduction is more likely to be successful if performed within 48 to 72 hours of the injury [11,16-18]. However, successful reductions have also been reported in cases treated up to 10 days post injury [4]. In cases where closed reduction fails or there are signs of neurovascular compromise, surgical stabilization may be required [3].

Although SCJ dislocations may appear minor, there remains a significant risk of catastrophic, life-threatening hemorrhage due to potential injury to mediastinal vascular structures. The involvement of a multidisciplinary team, including orthopedic, vascular, and anesthesia physicians, is crucial during the management of these injuries to ensure comprehensive care and mitigate the risks associated with potential complications [14,19]. In light of the high risk of catastrophic hemorrhage, it is imperative that sufficient typed and cross-matched blood, along with a rapid infusion system, be immediately available in the operating room [14,20].

## Conclusions

In conclusion, this case highlights the successful management of a posterior SCJ dislocation with a five-day delayed presentation using closed reduction. While early intervention is preferred, closed reduction can still be effective even when delayed, provided that there is no neurovascular compromise. Thorough imaging and surgical preparation are essential to ensure the safety of the patient, particularly due to the risk of mediastinal injury. The importance of a multidisciplinary approach, including vascular surgical support, and preoperative planning cannot be overstated in the management of these high-risk injuries. This case underscores the need for timely diagnosis, comprehensive imaging, and vigilant postoperative follow-up in patients with SCJ dislocations.
